# Identification and characterization of three chemosensory receptor families in the cotton bollworm *Helicoverpa armigera*

**DOI:** 10.1186/1471-2164-15-597

**Published:** 2014-07-15

**Authors:** Nai-Yong Liu, Wei Xu, Alexie Papanicolaou, Shuang-Lin Dong, Alisha Anderson

**Affiliations:** CSIRO Ecosystem Sciences, Black Mountain, Canberra, ACT 2601 Australia; Education Ministry Key Laboratory of Integrated Management of Crop Diseases and Pests, College of Plant Protection, Nanjing Agricultural University, Nanjing, 210095 China

**Keywords:** *Helicoverpa armigera*, Olfactory receptor, Gustatory receptor, Ionotropic receptor, Expression profile

## Abstract

**Background:**

Chemosensory receptors including olfactory receptors (ORs), gustatory receptors (GRs) and ionotropic receptors (IRs) play a central role in sensing chemical signals and guiding insect behaviours, and are potential target genes in insect pest control. The cotton bollworm *Helicoverpa armigera* is one of the most destructive pest species that can feed on over 200 different plant species. This diversity of host plants is likely linked to a complex chemosensory system. Here we built on previous work to characterize crucial chemosensory tissues linked to environmental interactions including larval antennae, larval mouthparts and larval fat bodies, as well as male and female adult heads, male and female adult tarsi, and female abdomens.

**Results:**

Using transcriptome sequencing, Trinity RNA-seq assemblies and extensive manual curation, we identified a total of 91 candidate chemosensory receptors (60 candidate ORs, 10 GRs and 21 IRs). Thirty-five of these candidates present full-length transcripts. First, we performed *in silico* differential expression analysis on different sequenced tissues. Further, we created extensive expression profiles using reverse transcription (RT)-PCR on a variety of adult and larval stages. We found that the expression profile of *HarmOR51* was limited to adult male antenna suggesting a role in mating that was further supported by a phylogenetic analysis clustering it into the pheromone receptor clade. HarmOR51 in calcium imaging analysis did not show responses to either of the two *H. armigera* sex pheromone components (*Z*9-16:Ald or *Z*11-16:Ald) inviting a future detailed study. In addition, we found four novel *HarmORs* (OR1, 53, 54 and 58) that appeared to be larvae-antennal specific. Finally, our expression profiling showed that four “divergent” *HarmIRs* (IR2, 7d.1, 7d.2 and 7d.3) were expressed in both adult and larval antennae, suggesting a functional divergence from their *Drosophila* homologues.

**Conclusions:**

This study explored three chemoreceptor superfamily genes using a curated transcriptomic approach coupled with extensive expression profiling and a more limited functional characterization. Our results have now provided an extensive resource for investigating the chemoreceptor complement of this insect pest, and meanwhile allow for targeted experiments to identify potential molecular targets for pest control and to investigate insect-plant interactions.

**Electronic supplementary material:**

The online version of this article (doi:10.1186/1471-2164-15-597) contains supplementary material, which is available to authorized users.

## Background

The chemosensory system is critical in guiding insect feeding, mating and oviposition behaviours [1]. Hair-like chemosensory sensilla distributed over the surface of chemosensory tissues including antennae, palps, mouthparts and tarsi, are used to detect chemical signals from the external environment [2, 3]. Chemosensory receptors of olfactory receptors (ORs) [4], gustatory receptors (GRs) [5] and ionotropic receptors (IRs) [6] are localized on the dendrite of chemosensory neurons housed in these sensilla. These receptors play a central role in helping insects detect chemical signals and regulate their behaviours [7], and are also important molecular targets for designing and developing new pest control strategies.

Even though *Drosophila melanogaster* is the main model insect for these genes [4–6], the availability of new genomic and transcriptomic sequences from other species is consistently extending both the phylogenetic coverage and the number of orthologs identified. The *D. melanogaster* genome hosts 60 OR genes that encode 62 ORs by alternative splicing [4]. Interestingly, insect ORs are highly diverse, share no sequence similarity and have an inverted membrane topology compared with mammalian OR genes, suggesting that insect ORs are not G-Protein Coupled Receptors (GPCRs) [8]. *Drosophila* GR genes have been classified into “CO_2_” [9], “sugar” [10], “GR43a-like” [11] and “bitter” clades [12]. GRs promote insect survival by detecting nutritious compounds and help avoid toxic ones [13]. Like ORs, these receptors share no sequence similarity with vertebrate GRs and their topology is inverted compared to the classic GPCRs [14, 15]. More recently, a variant sub-family of ionotropic glutamate receptors (iGluRs), the ionotropic receptor family, was identified as a new class of chemosensory receptors in *Drosophila* [6]. On the basis of their expression and sequence characteristics, *Drosophila* IRs were further distinguished into two sub-families: conserved “antennal IRs” involved in olfaction and species-specific “divergent IRs” that are expressed in other tissues including gustatory organs and may possibly be associated with gustation [16].

For this work we focused on the cotton bollworm (*Helicoverpa armigera* Hübner 1809; Lepidoptera: Noctuidae), an economically important species feeding on a wide range of host plants. *H. armigera* is one of the most polyphagous and cosmopolitan pest species with larvae that feed on numerous important cultivated crops such as cotton, peanuts, soybeans and maize. In a previous study, forty-seven OR genes were identified from *H. armigera* adult antennal transcriptomes but only one GR and 12 IR genes [17]. Only 22 full-length open reading frames were identified in this study, limiting any functional characterizations of these receptors. This previous transcriptome sequencing was restricted to adult antennae and did not consider other chemosensory tissues. Here, we conducted additional sequencing on other potential chemosensory tissues, including taste organs, not only in adults but also in larvae. Such transcriptomic data greatly improved the description of chemosensory receptors in *H. armigera* and will also assist in defining gene models for future genome studies.

Indeed, some chemosensory gene families have a low amino acid identity with their homologues and in our experience *de-novo* gene predictors have a limited capacity in annotating them. Even though genome sequencing will eventually provide a framework for full ascertainment, an extensive manual effort is required to curate complex genes such as GRs and IRs. Moreover, in this work we first used the expression profile and phylogenetic analysis to associate each gene with putative functions, and then characterized the functions of candidate pheromone receptors by the Sf9 calcium imaging technique. In addition, we outlined a path forward for an integrated study of the insect chemosensory system that can proceed solely based on transcriptomic information.

## Results

### Analysis of *H. armigera*transcriptome

We prepared 10 RNA-Seq libraries which after sequencing and quality control provided us with 239,276,681 read pairs of up to 100 bp in length (Additional file 1: Table S1). Our Trinity RNA-Seq assembly produced 68,100 contigs which included redundancy due to alternative transcription or high polymorphism and non-coding regions. After an initial protein prediction using a combination of a 5th order Markov chain and the Pfam database, we created a subset of 30,111 contigs with coding potential and annotated them with controlled vocabulary terms using the Uniprot database. These open reading frames are available via the NCBI BioProject PRJNA244590 (http://www.ncbi.nlm.nih.gov/bioproject/PRJNA244590). The full dataset, annotations such as Gene Ontology, Enzyme Classification, and visualizations can be explored at http://annotation.insectacentral.org/Liu2013. After extensive manual curation, we verified or reconstructed 91 chemosensory receptors.

### *In silico*differential expression profile of candidate *H. armigera*chemosensory receptors

To provide global gene expression profiles in different sequenced libraries, we surveyed the differential expression of all these open reading frames identified in this study. The complete result dataset is available at the CSIRO Data Portal (http://dx.doi.org/10.4225/08/535DB2C141C8A). As expected, all these genes (94 genes including *iGluRs*) were detected in at least one library. Most of these genes were expressed in male and/or female heads, where the antenna, proboscis and labial palp of crucial chemosensory organs, are located suggesting a functional role of these genes in olfaction or gustation. Specially, some genes appeared to be expressed only in male and/or female heads. For example, in the GR family *HarmGR1*; in the IR family *Harm*IR1, 41a, 75p.2 and 87a; and in the OR family five of seven pheromone receptors including the newly identified pheromone receptor *HarmOR51* were detected only in male heads. In summary, from 10 sequenced libraries we found the transcriptomic support of nearly all predicted chemosensory receptor genes.

### The *H. armigera*candidate olfactory receptors

**Figure 1:**
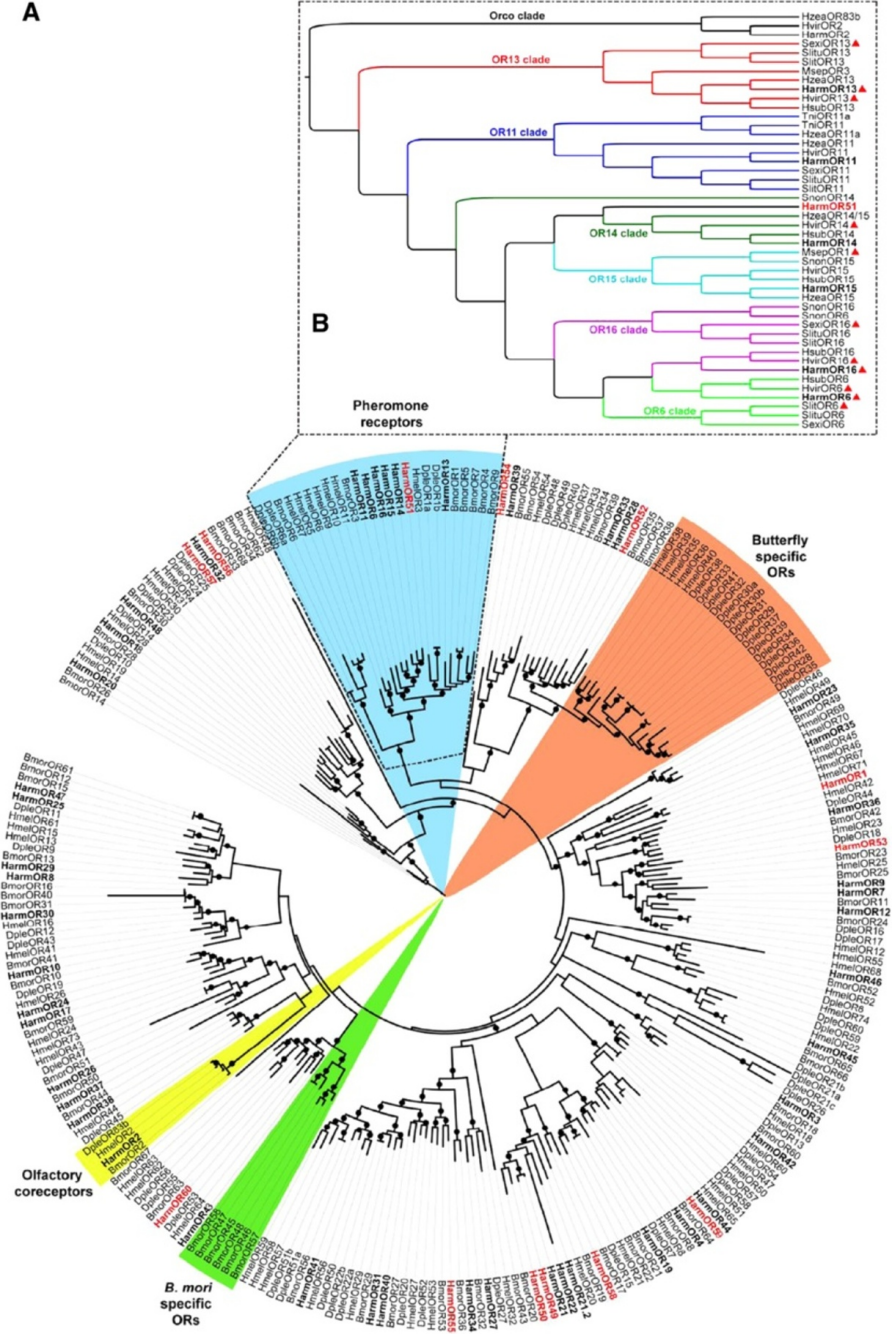
**Phylogenetic tree of putative** ***H. armigera*** **ORs with other insect ORs. (A)** The phylogenetic tree based on OR sequences from *H. armigera*, *B. mori*, *D. plexippus* and *H. melpomene*. **(B)** The phylogenetic tree based on PR sequences from 10 noctuid moths. The tree was constructed using PhyML under the JTT model of substitution with NNI topology search, based on an amino acid alignment by ClustalW2. Branch support was estimated using an approximate likelihood ratio test (Chi2) (circles: > 0.98). HarmORs are in bold and newly identified candidate HarmORs are highlighted with red letters. In Figure 1B, these PR genes with known ligands are labeled with red triangles, including *H. armigera* (this study, [18]), *H. virescens* [19, 20], *Mythimna separate* [21], *S. exigua* [22], and *S. littoralis* [23]. Bmor: *B. mori*; Dple: *D. plexippus*; Harm: *H. armigera*; Hmel: *H. melpomene*; Hvir: *H. virescens*; Hsub: *H. subflexa*; Hzea: *H. zea*; Msep: *Mythimna separate*; Sexi: *S. exigua*; Slit: *S. littoralis*; Slitu: *S. litura*; Snon: *S. nonagrioides*; Tni: *Trichoplusia ni*.

We identified 57 candidate ORs with 26 full-length sequences from *H. armigera* transcriptomes of larval antennae, mouthparts and fat bodies, male and female adult heads, male and female adult tarsi, and female abdomens. This included 44 previously identified ORs [17]. We named the genes as per the conventions followed by Liu *et al.* [17]. Integrating all public data, we found that the previously identified *HarmOR5* was actually a gustatory receptor based on a sequence search versus NCBI’s non-redundant database and a phylogenetic analysis, and therefore renamed it *HarmGR8* (Additional file 2: Table S2). We did not detect the previously identified HarmOR28, 33 and 37 genes in our transcriptome (Additional file 2: Table S2), so the previously published sequences were used for our phylogenetic analysis. These 60 *H. armigera* ORs were used for a phylogenetic clustering with 68 *Bombyx mori* ORs, 64 *Danaus plexippus* ORs and 69 *Heliconius melpomene* ORs (Figure 1A). In this analysis, sixteen *D. plexippus* ORs, five *H. melpomene* ORs (OR35, 36 and 38–40), and six *B. mori* ORs (OR45-48, 57 and 58) formed two monophyletic sub-families. One group of ORs formed by BmorOR1, 3–7 and 9, HarmOR6, 11, 13–16 and 51 and DpleOR1a, 1b, 6a and 6b was clustered with the pheromone receptor (PR) group. The novel candidate pheromone receptor HarmOR51 shared 68.4% and 62.9% identities to *Heliothis virescens* OR14 and HarmOR14, respectively. Further, a phylogenetic tree from noctuid PRs and co-receptors (Orco) allowed us to classify PRs into six main sub-families: the OR6, OR11, OR13, OR14, OR15 and OR16 clades (Figure 1B). The novel HarmOR51 was very close to the OR14 sub-family and the other six HarmORs were well clustered into each clade of noctuid PRs.

**Figure 2:**
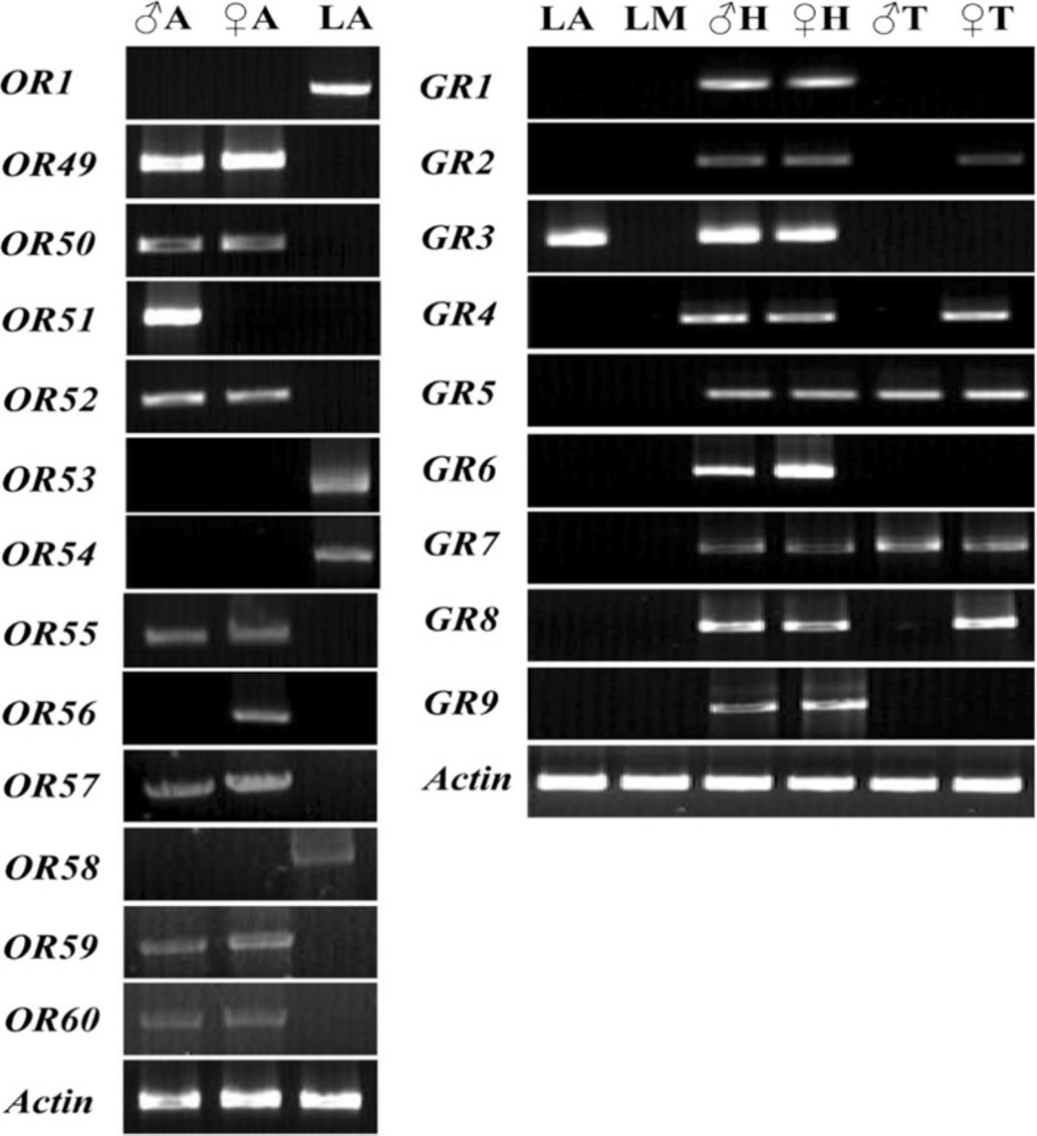
**Adult sex- and larval tissue- specific expression of putative** ***H. armigera*** **ORs and GRs.** Expression profiles of newly identified candidate HarmOR and GR genes were performed with gene-specific primers by RT-PCR. ♂A, male antenna; ♀A, female antenna; ♂H, male head; ♀H, female head; ♂T, male tarsi; ♀T, female tarsi; LA, larval antenna and LM, larval mouthpart. *Harmactin* gene was used as a control for all cDNA templates.

We used RT-PCR on adult and larval antennae to study the expression profiles of 13 newly identified *H. armigera* OR genes (*Harm*OR1, 49–60). The results showed that OR1, 53, 54 and 58 were detected only in larval antenna while the others were detected only in adult antenna, with the novel pheromone receptor *HarmOR51*, specifically expressed in the male antenna (Figure 2).

### The *H. armigera*candidate gustatory receptors

**Figure 3:**
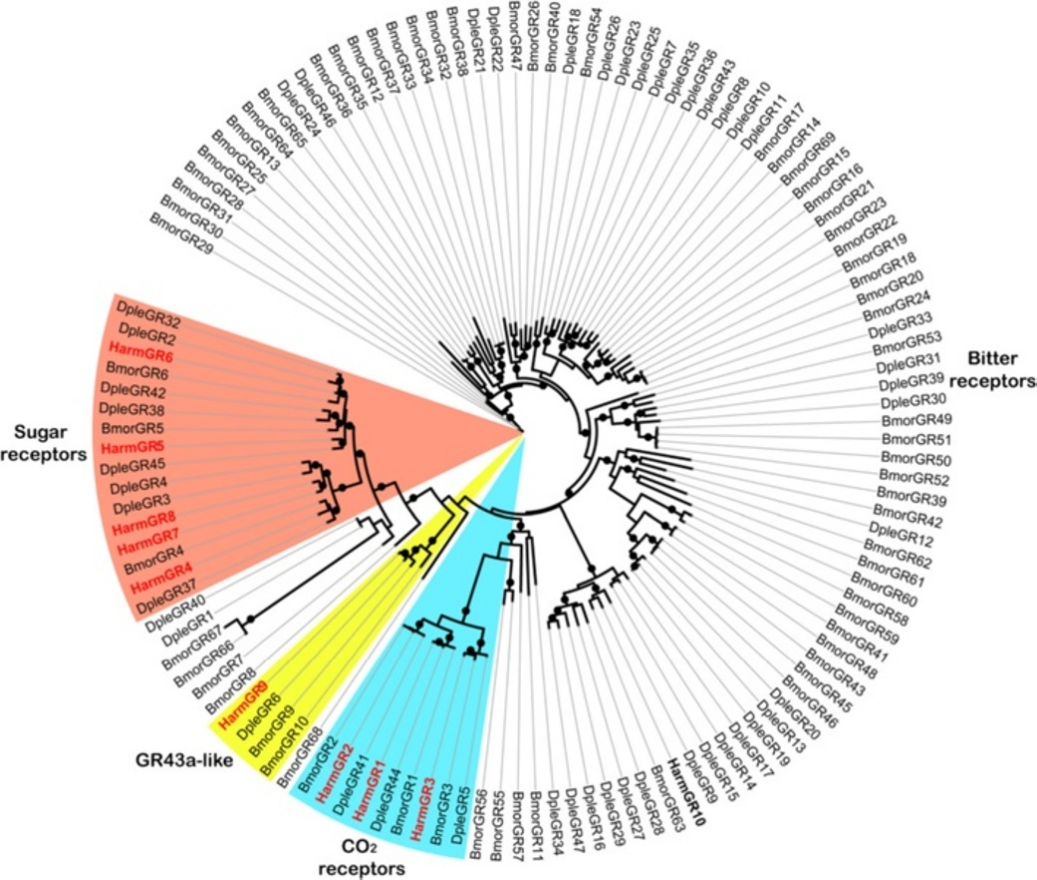
**Phylogenetic tree of putative** ***H. armigera*** **GRs with** ***B. mori*** **and** ***D. plexippus*** **GRs.** The tree was constructed using PhyML under the JTT model of substitution with NNI, based on an amino acid alignment by ClustalW2. Branch support was estimated using an approximate likelihood ratio test (Chi2) (circles: > 0.98). HarmGRs are in bold and newly identified candidate HarmGRs are highlighted with red letters. Bmor: *B. mori*; Dple: *D. plexippus*; Harm: *H. armigera*.

We identified nine candidate GRs from the *H. armigera* transcriptomes with HarmGR1-3 and HarmGR9 as full-length ORFs but the five others as partial sequences (Additional file 2: Table S2). We used a phylogenetic approach to name these genes. Previously reported *HarmGR1* that was not found in our transcriptome was renamed as *HarmGR10* following our identified *HarmGRs* in order to avoid a replicated name. A phylogenetic tree was built with 10 *H. armigera* GRs, 69 *B. mori* GRs and 47 *D. plexippus* GRs (Figure 3). HarmGR1-3 were orthologous to the silkworm “CO_2_” receptors, which shared ~76 to 90% identity to *B. mori* GR1-3, respectively. HarmGR4-8 and GR10 were members of the insect “sugar” receptor sub-family and shared ~10 to 65% identity to *B. mori* “sugar” GRs. HarmGR9, one member of the “GR43a-like” receptor sub-family, was orthologous to BmorGR9 (69% identity) and DmelGR43a (26% identity) receptors. None of the “bitter” receptors were found in our transcriptome sequences (Figure 3 and Additional file 2: Table S2).

Expression profiles were investigated with RT-PCR on larval antenna, larval mouthpart, male and female adult head, as well as male and female adult tarsi tissues. All of the sugar, CO_2_ and GR43a-like receptor genes were detected in the male and female adult heads, *Harm*GR5 and 7 were expressed in male and female adult tarsi, and *Harm*GR2, 4 and 8 were expressed only in female adult tarsi. Only *HarmGR3* was detected in larval antennae while none of GRs were detected in larval mouthparts (Figure 2).

### The *H. armigera*candidate ionotropic receptors

**Figure 4:**
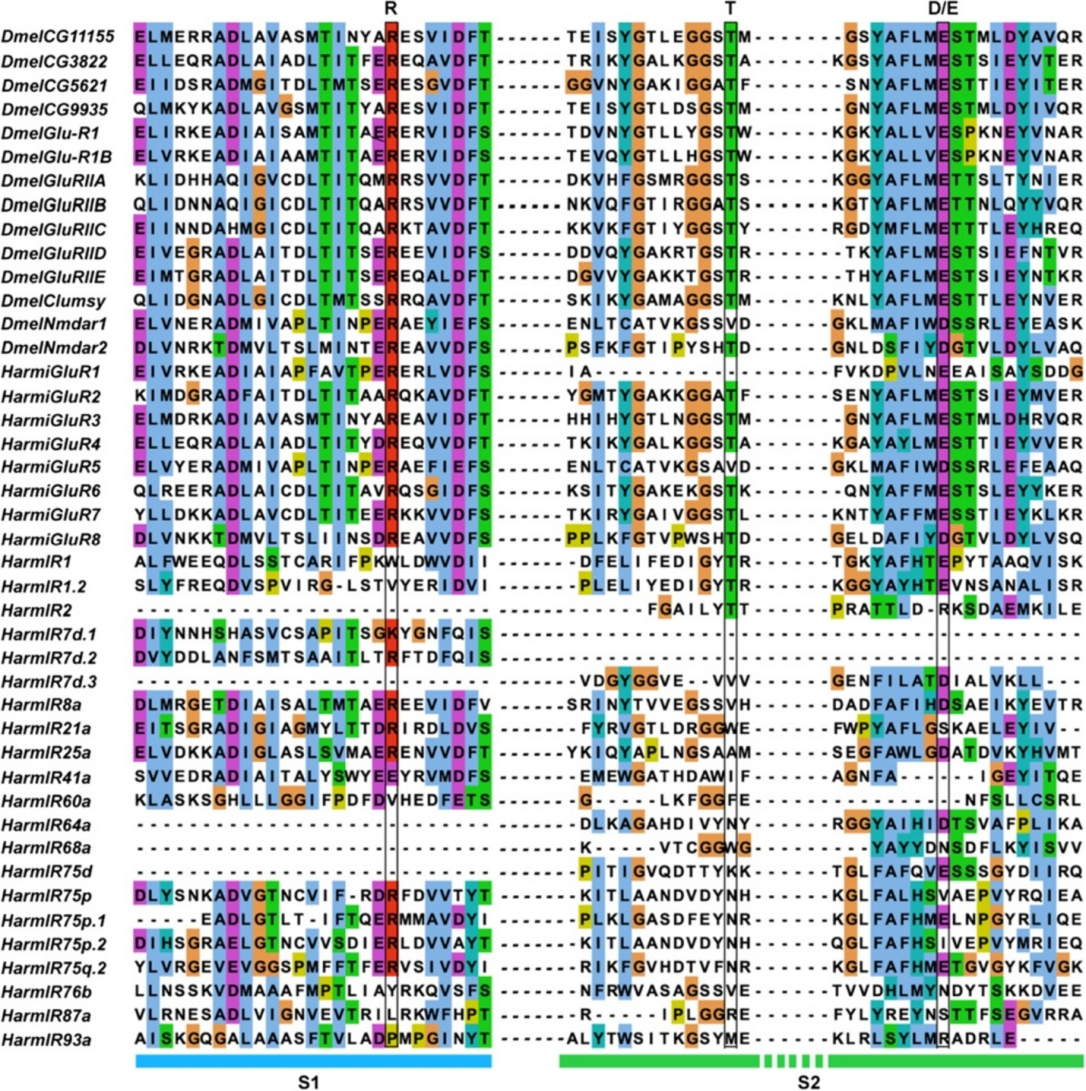
**Alignment of ligand-binding domains from putative** ***H. armigera*** **iGluRs and IRs with** ***D. melanogaster*** **iGluRs.** Three key ligand-binding residues (R, T and D/E) in iGluR family are boxed. S1 and S2 domains are marked with coloured boxes at the bottom.

We identified eight candidate iGluR and 20 IR genes from *H. armigera* transcriptomes including 11 of the 12 previously reported IRs [17]. *H. armigera* iGluRs were *de-novo* named using the Arabic numerals 1–8 and homologous *HarmIRs* were named based on the sequences of *D. melanogaster* and *B. mori*. One sequence did not present any similarities with reported IRs but retained their characteristic features, and thus was named *HarmIR2*. The amino acid sequences of eight candidate HarmiGluRs and 21 HarmIRs were aligned with *D. melanogaster* iGluRs. In HarmiGluR family with the exception of HarmiGluR1 and 5, a conserved amino acid profile in three key residues of ligand-binding domains was observed (arginine, threonine and aspartate/glutamate). The profile was not, however, conserved for the candidate HarmIRs, confirming their membership to the IR sub-family rather than the iGluR one (Figure 4). Further, we identified the full-length ORFs of two candidate co-receptors: HarmIR8a and 25a. They shared 49% and 63% amino acid identities with *D. melanogaster* IR8a and 25a, respectively, and showed a higher amino acid identity of over 70% with the IR8a and 25a of other lepidopteran species (*B. mori*, *Cydia pomonella*, *D. plexippus*, *Manduca sexta*, *Spodoptera littoralis* and *Sesamia nonagrioides*). In addition, the full-length ORFs of a Lepidoptera-specific IR87a solely expressed in adult antennae [24] and a candidate receptor IR76b involved in salt-taste coding in *D. melanogaster* [25] were also found in our data (Additional file 2: Table S2).

**Figure 5:**
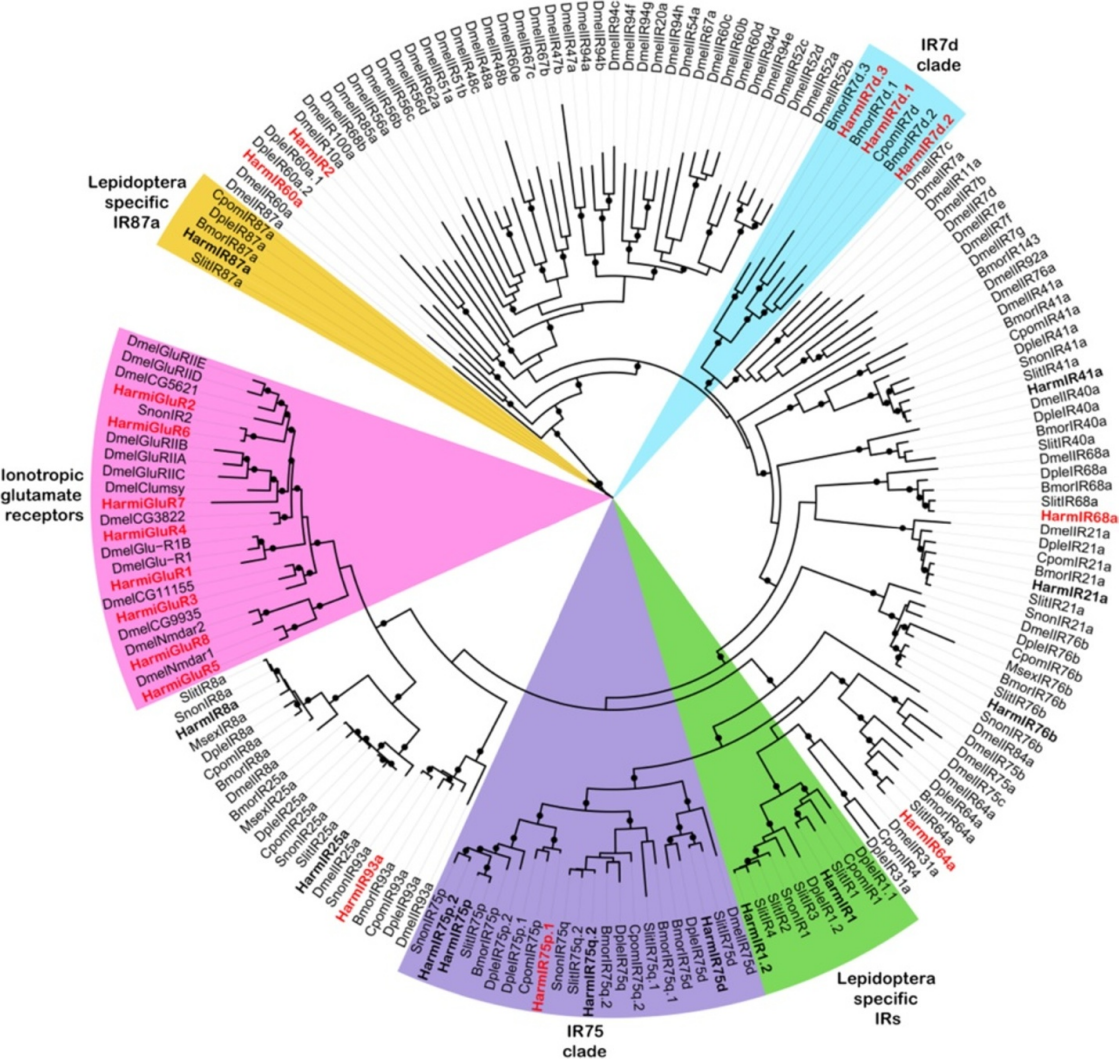
**Phylogenetic tree of putative** ***H. armigera*** **iGluRs and IRs with** ***D. melanogaster*** **iGluRs and IRs and other lepidopteran IRs.** The tree was built using PhyML with the JTT model of substitution with NNI. Branch support was estimated by approximate likelihood ratio test (Chi2) (circles: > 0.98). HarmIRs are in bold and in this study newly identified candidate HarmIRs are highlighted with red letters. Bmor, *B. mori*; Cpom, *C. pomonella*; Dmel, *D. melanogaster*; Dple, *D. plexippus*; Harm, *H. armigera*; Msex, *M. sexta*, Slit, *S. littoralis* and Snon, *S. nonagrioides*.

The phylogenetic tree of HarmiGluRs and IRs was constructed with other lepidopteran IRs and *D. melanogaster* iGluRs and IRs (Figure 5). We annotated nine new candidate *HarmIR* genes, five of which (IR60a, 64a, 68a, 75p.1 and 93a) were well clustered into conserved antennal IRs and the remaining ones (IR2, 7d.1, 7d.2 and 7d.3) were classified into species-specific divergent IRs. HarmIR7d.1, 7d.2 and 7d.3 formed a sister clade to the *B. mori* IR7d clade. A new divergent HarmIR2 was not classified into any lepidopteran IR clades, but was clustered into *Drosophila* divergent IRs. In addition, two large sub-families (IR7d and IR75 clades) and a potentially specific IR group [24, 26] in lepidopteran species were observed (Figure 5).

**Figure 6:**
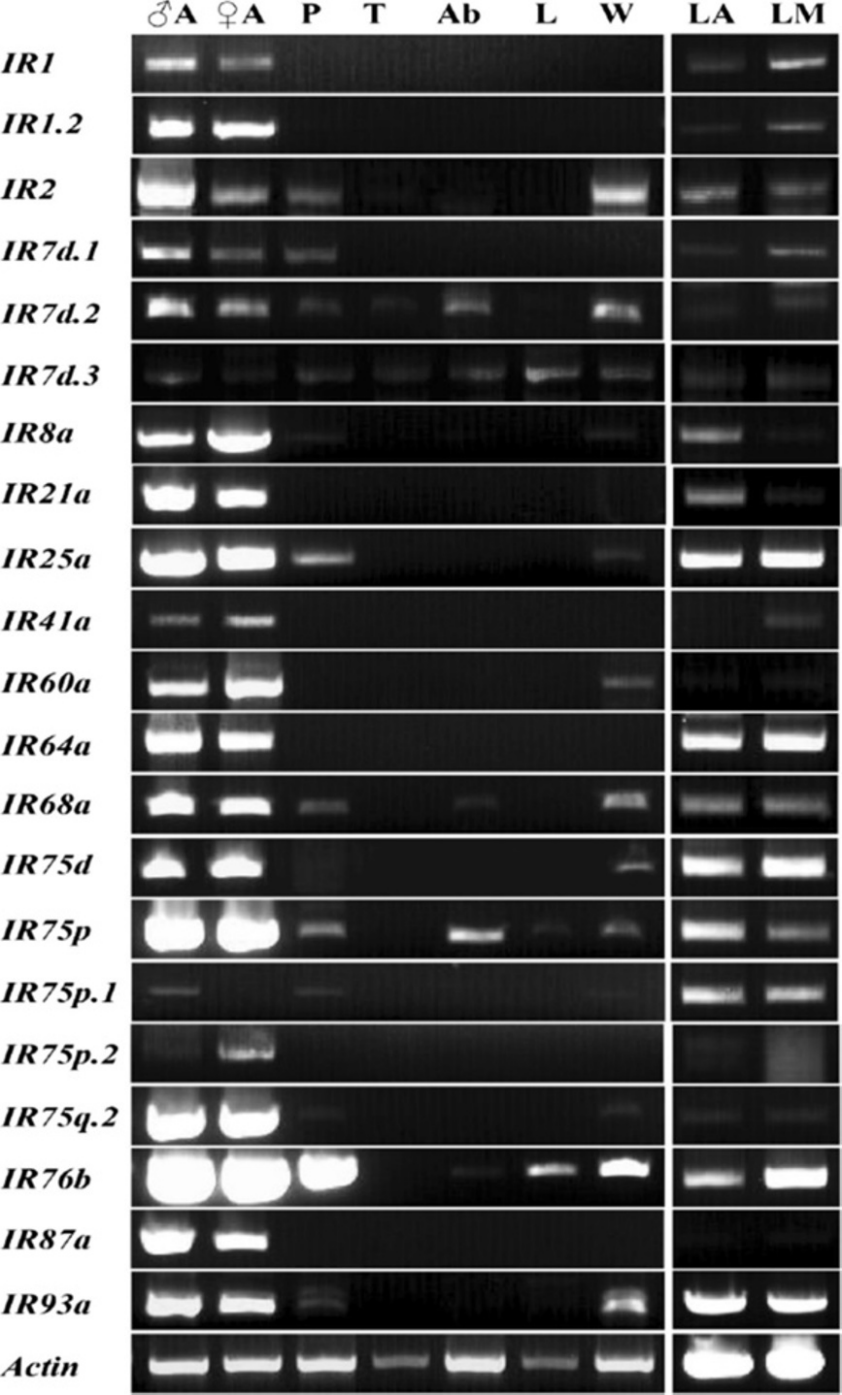
**Adult and larval tissue-specific expression of putative** ***H. armigera*** **IRs.** Expression profiles of all putative HarmIR genes were performed with gene-specific primers by RT-PCR. ♂A, male antenna; ♀A, female antenna; P, mixed proboscises; T, mixed thoraxes; Ab, mixed abdomens; L, mixed legs; W, mixed wings; LA, larval antenna and LM, larval mouthpart. *Harmactin* gene was used as a control for all cDNA templates.

RT-PCR expression profiles were built for a number of adult tissues as well as larval antennae and mouthparts. In the adult tissues, the results showed that all of these *HarmIR* genes were strongly expressed in antennae of both sexes except for *Harm*IR75p.1 and 75p.2 exhibiting only a low level of expression. The *HarmIR87a* gene appeared to be specifically expressed in adult antennae. Additionally, there were as many as 12 *HarmIRs* expressed in proboscises or wings. Most *HarmIR* genes were detected in larval antennae and mouthparts, with only some exhibiting no or low expression, like *Harm*IR1.2, 7d.2, 41a, 60a, 75p.2 and 87a (Figure 6).

### Functional studies of candidate *H. armigera*pheromone receptors in Sf9 cells

**Figure 7:**
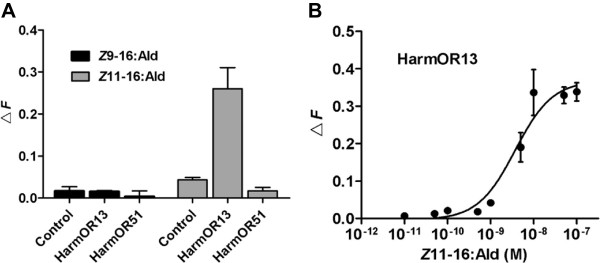
**Calcium imaging analysis of** ***H. armigera*** **OR13 and OR51 to sex pheromone components,** ***Z*** **11-16:Ald and** ***Z*** **9-16:Ald. (A)** Responses of HarmOR13 and OR51 to two sex pheromone components (10^-7^ M). HarmOR13 showed significant responses to *Z*11-16:Ald but not *Z*9-16:Ald, while HarmOR51 did not show any responses to either *Z*11-16:Ald or *Z*9-16:Ald. **(B)** Dose-dependent response curve of HarmOR13 to *Z*11-16:Ald. Error bars represent standard errors (*N* = 3).

We used Sf9 cells coupled with the calcium imaging technique [27] to assess the activity of HarmOR13 and 51 to the major sex pheromone component (*Z*)-11-hexadecenal (*Z*11-16:Ald) as well as one minor component (*Z*)-9-hexadecenal (*Z*9-16:Ald) (Figure 7). HarmOR13 showed a significant response to *Z*11-16:Ald but not *Z*9-16:Ald, as seen in previous studies using the *Xenopus* system [18]. Moreover, HarmOR13 showed an apparent dose–response to *Z*11-16:Ald with EC_50_ value of 3.71 × 10^-9^ M. HarmOR51 did not show any responses to either *Z*9-16:Ald or *Z*11-16:Ald.

## Discussion

In this work we built upon previous studies to investigate an array of tissues that play an important role in insect-plant interactions [28, 29]: larval antennae, larval mouthparts, larval fat bodies, as well as female abdomens, adult tarsi and adult heads with antenna, proboscis and labial palp. Our research interest primarily focused on three crucial repertoires of chemosensory receptor superfamilies (ORs, GRs and IRs) due to their significance as potential target genes for designing and developing new pest control strategies and addressing the insect host-plant interactions. After the extensive sequencing and assembly using Trinity RNA-Seq, we manually curated every transcriptome so that the community now has accessed to a repertoire of 60 ORs, 10 GRs and 21 IRs. We found that *H. armigera* has more IRs than in any other lepidopteran species reported to date [30]. Further, nine *H. armigera* GRs were found in the transcriptomic data, although gustatory receptors have been reported to exhibit extremely low expression in gustatory organs [5, 31]. We were also able to fully reconstruct the transcripts of the newly predicted *H. armigera* chemosensory receptors from sequenced libraries.

In addition to verifying our transcript reconstructions, an extensive sex-, tissue- and stage-specific expression profile using RT-PCR for the newly identified ORs and GRs, as well as all the IRs allowed us to further characterize these genes. We found four larvae-specific *HarmORs* which may be involved in larvae-specific behaviours. It has been reported that *BmorOR56*, expressed in silkworm larvae, mediated larval responses to *cis*-jasmone that may be emitted from mulberry leaves as an attractant [28]. These larvae-specific *HarmORs* may be candidate targets for future larval control. Phylogenetic analysis showed that our newly identified HarmOR51 was clustered into the pheromone receptor sub-family, and shared a high identity to other noctuid PRs. Thus we believe that it is possibly a new candidate of *H. armigera* pheromone receptors, leading to a total of seven pheromone receptors reported in a lepidopteran species for the first time.

Further, we used the Sf9 calcium technique to measure the binding of HarmOR13 and 51 to the *H. armigera* sex pheromone components (*Z*11-16:Ald or *Z*9-16:Ald). This system is likely to more closely resemble *in vivo H. armigera* olfactory receptor functions, since insect Sf9 cells are derived from a lepidopteran species *S. frugiperda*, and express a native co-receptor (Orco, a highly-conserved OR across different insect taxa) to assist HarmORs in ligand detection. We identified a strong response of HarmOR13 to *Z*9-16:Ald, as seen in another functional study [18]. The HarmOR51 receptor, however, did not elicit a response to either component tested, and needs to be investigated in a comprehensive future analysis. Although our calcium imaging did not help identify the ligands for HarmOR51, it did work very well in the analysis of HarmOR13 to *Z*11-16:Ald, suggesting that the assay could be applied in future analyses to seek potential ligands for other ORs.

Four novel divergent *HarmIRs* were detected in both larval and adult antennae, suggesting a different function to the *Drosophila* divergent IRs that are not expressed in the antennae of any stages [16]. Our extensive expression profile showed that most *HarmIRs* were expressed in antennae (at least 19 in adult and 18 in larva), providing a more extensive repertoire of antennal IRs compared to 16 of *D. melanogaster* [6, 30]. Some *HarmIRs* were detected in adult proboscis and larval mouthpart, indicating that these IRs may also have a gustatory function as previously indicated in *D. melanogaster* [16] and *S. littoralis* [24].

## Conclusions

In this study, we sequenced crucial chemosensory tissues, and utilized an integrated approach to probe three chemosensory receptor repertoires in our transcriptome. We identified a total of 91 chemosensory receptor genes comprised of 60 ORs, 10 GRs and 21 IRs (an increase of 13 ORs, nine GRs and nine IRs compared to the previous work). In particular, the novel *HarmOR51*, the orthologous genes of which have been never found in any other lepidopteran species, was identified as a new potential pheromone receptor. We also carried out, for the first time, an extensive sex-, tissue- and stage- expression profile for all *H. armigera* IRs that showed a widespread expression of *HarmIRs* in non-antennal tissues, implying that in this species IRs have a more complex function. Together, this study greatly complements the information of chemosensory receptors in *H. armigera*, and shows the utility of combining transcriptomic, phylogenetic and functional assays to elucidating the function and evolution of the insect chemosensory system.

## Methods

### Insect rearing and tissue collection

*H. armigera* were fed an artificial diet in the laboratory of CSIRO under conditions described previously [32]. Approximately 200 larval antennae, 200 larval mouthparts and two larval fat bodies were dissected from fifth instar larvae. Five heads with antenna, proboscis and labial palp, 50 tarsi and two female abdomens were collected from 1 to 5-day-old male and female moths. In expression profile studies, all adult tissues were collected from 3-day-old male and female moths. All collected tissues were immediately stored in *RNAlater* (Invitrogen, USA).

### Extraction of total RNA and first-strand cDNA synthesis

Total RNA was purified using RNeasy (Qiagen, USA) or RNAqueous (Ambion, USA) kits according to the manufacturer's protocol. The purified RNA was then treated with *DNase*I (Ambion, USA) at 37°C for 30 min along with the manufacturer’s protocol, quantified and qualified by NanoDrop ND-2000 (Thermo Scientific, USA) as well as 2100 Bioanalyzer (Agilent, USA).

cDNA templates were prepared from purified RNA samples of larval and adult tissues using SuperScript™ III Reverse Transcriptase (Invitrogen, USA), according to the manufacturer's manual. Briefly, the first-strand cDNA was synthesized with Oligo(dT)_20_ primer at 65°C for 5 min, and then at 50°C for 60 min. The reaction was stopped at 70°C for 15 min. The templates were stored at -20°C until use.

### Sequencing and transcriptome analysis

The prepared RNA samples from larval antennae, larval mouthparts, larval fat bodies, male adult tarsi, female adult tarsi and female abdomens were sent to BGI-Tech for generating Paired-End Illumina TruSeq libraries and sequencing with an Illumina HiSeq 2000 platform. To help identify the chemosensory genes which are lowly expressed, we further sequenced the sample libraries of male and female adult heads that were treated by using the Duplex‒Specific thermostable nuclease (DSN) enzyme (Evrogen) according to the DSN normalization protocol (Illumina). The RNA samples from male and female adult heads as well as male and female adult heads with DSN treatment were used to prepare Paired-End Illumina TruSeq libraries according to the manufacturer’s protocol and sent to ACRF Biomolecular Resource Facility for Illumina sequencing service (http://brf.anu.edu.au/).

All RNAseq data were then pre-processed using the default settings of Just_Preprocess_My_Reads (http://justpreprocessmyreads.sourceforge.net) which conducted a mild quality control and trimming, pooled and assembled using Trinity-RNASeq using the default settings as per [33]. Trinity RNA-Seq is highly capable of overcoming quality and polymorphism issues due to bubble popping algorithms in each of the three modules, Inchworm, Chrysalis and Butterfly. Open reading frames (ORFs) were predicted using the TranscriptDecoder software in Trinity-RNASeq (http://sourceforge.net/projects/transdecoder) with the Pfam option. The raw sequence data and coding sequences of the transcriptome assembly have been deposited in the National Center for Biotechnology Information (NCBI) (accession number: PRJNA244590; http://www.ncbi.nlm.nih.gov/bioproject/PRJNA244590).

*In silico* expression profiles were generated using DEW (http://dew.sourceforge.net/) which is an automated pipeline that: 1) used Bowtie2 [34] to align the reads of each library against a sequence file comprised of our assembly; 2) post-processed the alignments with eXpress [35] to account for isoforms and paralogues; 3) performed Trimmed Mean Normalization using edger and estimated Fragments Per Kilobase Per Million reads (FPKM) [36]; and 4) visualized the results using ggplot2 [37] and canvasXpress (http://canvasxpress.org/). From these data, certain cutoffs were used to make a true/false decision on whether a gene was expressed: a gene had to have at least four RNA-Seq reads aligned against it and covering at least 0.30 of its length. Functional annotations were performed and visualised using Just_Annotate_My_Proteins (JAMp; http://sourceforge.net/projects/jamps) which is an automated pipelines that 1) used HHblits [38] to search against Hidden Markov Models derived from the curated Uniprot archive; 2) assigned controlled vocabulary terms (e.g. GO, KEGG etc.) linked to a Uniprot accession only if an actual experiment provides evidence (i.e. we did not use any ‘inference via electronic similarity’ evidence); 3) visualized them using ExtJS4 and canvasXpress. Up to 10 HHblits alignments that passed the following JAMp cutoffs were used: homology probability: >= 80%; homology e-value: <= 1e-10; homology p-value: <= 1e-12; HHblits score: >= 70. These assemblies were then subsequently mined and curated using Geneious 5 to create a non-redundant set of the *Helicoverpa* chemosensory genes.

### Identification of candidate chemosensory receptors

TBLASTN searches were carried out with previously identified *H. armigera*, *B. mori* and *Drosophila melanogaster* ORs [4, 28], GRs [5, 39] and IRs [6, 17]. In order to verify these receptors, amino acid sequences of all identified candidate receptors were searched against NCBI non-redundant protein database using BLASTX based on the identity and similarity to orthologous genes from other insect species. These gene sequences were finally compared to previously described chemosensory receptors [17] to identify novel genes. All newly identified *H. armigera* OR, GR, as well as iGluR and IR amino acid sequences are available in GenBank as ACCESSION (Additional file 2: Table S2).

### Sequence and phylogenetic analysis

Alignments of amino acid sequences were performed by ClustalW2 [40] and were visualized by Jalview 2.7 [41]. Phylogenetic trees were constructed by PhyML [42] based on Jones-Taylor-Thornton (JTT) model with Nearest Neighbour Interchange (NNI). Branch support was estimated by approximate likelihood ratio test (Chi2). In the OR data set, we selected the ORs with available genomic databases from *B. mori* [28], *D. plexippus* [43] and *H. melpomene* [44]. Due to the small number of HarmGRs, we only selected GRs from two species, a moth *B. mori* [39] and a butterfly *D. plexippus* [43]. In the IR data set, we selected IRs from lepidopteran species including *B. mori* [16], *C. pomonella* [45], *D. plexippus* [43], *M. sexta* [46], *S. littoralis* [26] and *S. nonagrioides* [47] but also IRs and iGluRs from a model insect *D. melanogaster* [16]. Trees were viewed and edited using iTol [48, 49]. Networks using the protein identity were generated using custom scripts and analyzed with CytoScape [50] and clusterMarker [51].

### RT-PCR

To verify expression of candidate *Harm*OR, GR and IR genes identified from our transcriptome and to investigate sex- and tissue- expression profiles, RT-PCR was performed using gene-specific primers (Additional file 3: Table S3) as follows: 95°C for 3 min; 35 cycles at 95°C for 30 s, 60°C for 30 s and 72°C for 40 s; and final extension at 72°C for 7 min. *H. armigera actin* gene (GenBank accession number: X97614) was used as control to check the quality of cDNA templates. For each RT-PCR amplification, negative controls using sterile water as the template were performed. Each gene PCR reaction was repeated at least twice. PCR products were analysed using 1.5% agarose gels. The expected products of the genes randomly selected were sequenced to confirm the identity with their original sequence identified.

### Calcium imaging

The sex pheromone *Z*11-16:Ald was purchased from Sigma-Aldrich (95% purify), and another component *Z*9-16:Ald was synthesized from Yick-Vic Chemicals & Pharmaceulicals (Hong Kong, China) (>95% purify). In the calcium imaging experiment, sex pheromones were dissolved in dimethyl sulfoxide (DMSO) as 1 M stock solution and stored at -20°C according to previously reported protocols [52]. For dose-dependent assays, pheromone solutions were diluted from stock solution to the desired concentration with HBSS buffer [14].

HarmOR13 and HarmOR51 ORFs were cloned with 35 cycles and an annealing temperature of 60°C using gene-specific primers (Additional file 3: Table S3). The PCR products were ligated into pBluescript vector, previously digested by EcoRV. Positive clones were sequenced. The verified plasmids were digested with KpnI and SacII for HarmOR13, and HindIII and XbaI for HarmOR51. And the digested target genes were ligated into PIB/V5-His vector, previously digested by the same enzymes. The constructed plasmids were further sequenced to confirm the orientation and sequence. Next, Sf9 cells were plated into 12-well plates and left to settle for about 20 min. Finally, the cells were transfected with 500 ng of plasmid construct PIB/HarmORs or PIB/V5-His vector (negative control) and 3 μL of Fugene HD transfection reagent (Promega, USA) in 100 μL of medium per well. After 48 h post-transfection, the methods of cell treatment and functional assay were the same as previously described [14, 53]. All data were analyzed by GraphPad 5.0.

### Availability of supporting data

All supporting data are included as additional files. The data of transcriptome assembly were deposited in the National Center for Biotechnology Information (NCBI) (accession number: PRJNA244590; http://www.ncbi.nlm.nih.gov/bioproject/PRJNA244590). The transcriptome dataset and annotations can be found at http://annotation.insectacentral.org/Liu2013. The dataset of gene expression profiles is available at the CSIRO Data Portal (http://dx.doi.org/10.4225/08/535DB2C141C8A). Phylogenetic data were deposited in the CSIRO Data Portal (http://dx.doi.org/10.4225/08/538FD6C5C9897).

## Electronic supplementary material

Additional file 1: Table S1: Overview of RNASeq libraries sequenced for this study. (XLSX 11 KB)

Additional file 2: Table S2: The identified putative *H. armigera* ORs, GRs, iGluRs and IRs. (PDF 26 KB)

Additional file 3: Table S3: Primers for RT-PCR experiments of *H. armigera* OR, GR and IR genes and calcium imaging analysis of *H. armigera* OR genes. (PDF 8 KB)
