# Supplementary material for: Identification and characterization of three chemosensory receptor families in the cotton bollworm Helicoverpa armigera
Source: BMC Genomics. 2014 Jul 15;15(1):597. doi: 10.1186/1471-2164-15-597 (PMC4112213; doi:10.1186/1471-2164-15-597)
Supplement: Supplementary file 2 — Additional file 2: Table S2: The identified putative H. armigera ORs, GRs, iGluRs and IRs. (PDF 26 KB) [file 12864_2013_6286_MOESM2_ESM.pdf]

Table S2

Gene	ORF (aa)	Accession no.	BLASTx hit (Reference/Name/Species)	E value	Identity (%)	Full-length
OR1	116	KF768656	XP_004933118 putative odorant receptor 85b-like [*Bombyx mori*]	3e-66	89	N
OR2	473	KF768657	AAX14773 odorant receptor Or83b [*Helicoverpa zea*]	0	100	Y
OR3	200	KF768658	CAD31852 putative chemosensory receptor 3 [*Heliothis virescens*]	1e-133	94	N
OR4	181	KF768659	NP_001157209 olfactory receptor 8 [*Bombyx mori*]	2.3e-79	81	N
OR5			Identified as GR8			
OR6	425	KF768660	CAG38117 putative chemosensory receptor 16 [*Heliothis virescens*]	0	71	Y
OR7	359	KF768661	CAD31853 putative chemosensory receptor 7 [*Heliothis virescens*]	0	97	N
OR8	396	KF768662	CAD31949 putative chemosensory receptor 8 [*Heliothis virescens*]	0	74	Y
OR9	265	KF768663	CAD31950 putative chemosensory receptor 9 [*Heliothis virescens*]	8e-167	85	N
OR10	250	KF768664	CAG38111 putative chemosensory receptor 10 [*Heliothis virescens*]	4e-115	98	N
OR11	430	KF768665	CAG38112 putative chemosensory receptor 11 [*Heliothis virescens*]	0	95	Y
OR12	408	KF768666	CAG38113 putative chemosensory receptor 12 [*Heliothis virescens*]	0	90	Y
OR13	425	KF768667	ACS45307 candidate odorant receptor 1 [*Helicoverpa assulta*]	0	95	Y
OR14	314	KF768668	CAG38115 putative chemosensory receptor 14 [*Heliothis virescens*]	0	83	N
OR15	440	KF768669	CAG38116 putative chemosensory receptor 15 [*Heliothis virescens*]	0	83	Y
OR16	422	KF768670	CAG38117 putative chemosensory receptor 16 [*Heliothis virescens*]	0	89	Y
OR17	396	KF768671	CAG38118 putative chemosensory receptor 17 [*Heliothis virescens*]	0	93	N
OR18	398	KF768672	ADN03364 olfactory receptor 18 [*Helicoverpa assulta*]	0	99	Y
OR19	356	KF768673	CAG38120 putative chemosensory receptor 19 [*Heliothis virescens*]	3e-174	69	N
OR20	393	KF768674	ADN03363 olfactory receptor 20, partial [*Helicoverpa assulta*]	0	92	Y
OR21	188	KF768675	CAG38122 putative chemosensory receptor 21 [*Heliothis virescens*]	6e-113	88	N
OR21-2	403	KF768676	CAG38122 putative chemosensory receptor 21 [*Heliothis virescens*]	6e-89	38	Y

OR22	415	KF768677	AFC91742 putative odorant receptor OR34 [*Cydia pomonella*]	6e-66	36	Y
OR23	382	KF768678	AEF32141 odorant receptor [*Spodoptera exigua*]	0	77	N
OR24	391	KF768679	NP_001091790 candidate olfactory receptor [*Bombyx mori*]	1.04e-148	65	Y
OR25	387	KF768680	DAA05974 TPA_exp: odorant receptor 15 [*Bombyx mori*]	1.25e-120	46	Y
OR26	408	KF768681	AGG08876 putative olfactory receptor 51 [*Spodoptera litura*]	0	82	Y
OR27	355	KF768682	BAH66328 olfactory receptor [*Bombyx mori*]	1.22e-102	52	N
OR28[a]		[c]	Not identified			
OR29	404	KF768683	NP_001166603 olfactory receptor 13 [*Bombyx mori*]	8e-105	48	N
OR30	288	KF768684	EHJ70341 olfactory receptor 16 [*Danaus plexippus*]	2e-150	70	N
OR31	395	KF768685	NP_001166894 olfactory receptor 29 [*Bombyx mori*]	0	70	Y
OR32	349	KF768686	NP_001103623 olfactory receptor 33 [*Bombyx mori*]	2.51e-114	42	N
OR33[a]		[c]	Not identified			
OR34	233	KF768687	DAA05992 TPA_exp: odorant receptor 36 [*Bombyx mori*]	1.76e-66	53	N
OR35	299	KF768688	NP_001091818 olfactory receptor 42 [*Bombyx mori*]	9.84e-27	31	N
OR36	250	KF768689	NP_001091818 olfactory receptor 42 [*Bombyx mori*]	6.53e-177	65	N
OR37[a]		[c]	Not identified			
OR38	264	KF768690	NP_001166607 olfactory receptor 44 [*Bombyx mori*]	0	78	N
OR39	388	KF768691	AFC91744 putative odorant receptor OR36 [*Cydia pomonella*]	2.16e-82	37	Y
OR40	401	KF768692	NP_001166893 olfactory receptor 27 [*Bombyx mori*]	6.95e-153	58	Y
OR41	402	KF768693	NP_001166617 olfactory receptor 56 [*Bombyx mori*]	0	75	Y
OR42	442	KF768694	NP_001155301 olfactory receptor 60 [*Bombyx mori*]	0	69	Y
OR43	396	KF768695	NP_001166620 olfactory receptor 63 [*Bombyx mori*]	3.8e-137	51	N
OR44	415	KF768696	NP_001166621 olfactory receptor 64 [*Bombyx mori*]	1.22e-93	53	Y
OR45	429	KF768697	AFC91732 putative odorant receptor OR24 [*Cydia pomonella*]	4.65e-158	59	N
OR46	448	KF768698	NP_001116817 olfactory receptor-like [*Bombyx mori*]	2.84e-161	68	Y

OR47	191	KF768699	DAA05974 TPA_exp: odorant receptor 15 [*Bombyx mori*]	1.18e-124	46	N
OR48	283	KF768700	BAH66327 olfactory receptor [*Bombyx mori*]	5.16e-82	51	N
OR49	330	KF768701	CAG38122 putative chemosensory receptor 21 *[Heliothis virescens*]	0	83	N
OR50	397	KF768702	CAG38122 putative chemosensory receptor 21 [*Heliothis virescens*]	2.76e-136	50	N
OR51	440	KF768703	CAG38115 putative chemosensory receptor 14 [*Heliothis virescens*]	0	68	Y
OR52	419	KF768704	NP_001103476 olfactory receptor 35 [*Bombyx mori*]	2.41e-160	53	Y
OR53	401	KF768705	DAA05981 TPA_exp: odorant receptor 23 [*Bombyx mori*]	3.70e-73	42	Y
OR54	243	KF768706	NP_001166614 olfactory receptor 49 [*Bombyx mori*]	1.61e-90	62	N
OR55	215	KF768707	AFC91736 putative odorant receptor OR28 [*Cydia pomonella*]	3.35e-171	56	N
OR56	369	KF768708	BAH66329 olfactory receptor [*Bombyx mori*]	8.39e-73	36	N
OR57	404	KF768709	NP_001103623 olfactory receptor 33 [*Bombyx mori*]	4.65e-93	36	N
OR58	133	KF768710	NP_001157210 olfactory receptor 17 [*Bombyx mori*]	3.43e-122	48	N
OR59	238	KF768711	NP_001166621 olfactory receptor 64 [*Bombyx mori*]	1.27e-94	54	N
OR60	391	KF768712	NP_001166620 olfactory receptor 63 [*Bombyx mori*]	8.77e-180	64	Y
GR1	467	KF768713	XP_004932762 gustatory and odorant receptor 22-like [*Bombyx mori*]	0	85	Y
GR2	433	KF768714	XP_004932263 gustatory and odorant receptor 22-like [*Bombyx mori*]	0	89	Y
GR3	465	KF768715	EHJ78216 gustatory receptor 24 [*Danaus plexippus*]	0	83	Y
GR4	359	KF768716	XP_004923090 putative gustatory receptor 64a-like [*Bombyx mori*]	8e-144	62	N
GR5	489	KF768717	CAD31850 putative chemosensory receptor 1 [*Heliothis virescens*]	8e-127	47	N
GR6	296	KF768718	CAD31850 putative chemosensory receptor 1 [*Heliothis virescens*]	0	96	N
GR7	316	KF768719	CAD31947 putative chemosensory receptor 5 [*Heliothis virescens*]	2e-153	65	N
GR8	309	KF768720	CAD31947 putative chemosensory receptor 5 [*Heliothis virescens*]	0	91	N
GR9	465	JX970522	CAD31946 putative chemosensory receptor 4 [*Heliothis virescens*]	0	98	Y
GR10[a]		[c]	Not identified			
iGluR1	712	KF768730	XP_004929956 PREDICTED: glutamate receptor 1-like [*Bombyx mori*]	0	79	N

iGluR2	938	KF768731	XP_004932731 PREDICTED: glutamate receptor ionotropic kainate 3-like [*Bombyx mori*]	0	76	Y
iGluR3	566	KF768732	EHJ65357 hypothetical protein KGM_17294 [*Danaus plexippus*]	0	82	N
iGluR4	902	KF768733	XP_004932732 PREDICTED: glutamate receptor ionotropic kainate 2-like [*Bombyx mori*]	0	95	Y
iGluR5	932	KF768734	EHJ78211 putative NMDA-type glutamate receptor 1 [*Danaus plexippus*]	0	94	Y
iGluR6	900	KF768735	XP_001655460 ionotropic glutamate receptor subunit ia [*Aedes aegypti*]	0	40	Y
iGluR7	924	KF768736	EHJ66741 hypothetical protein KGM_16051 [*Danaus plexippus*]	0	59	Y
iGluR8	929	KF768737	EHJ66761 putative glutamate receptor, ionotropic, n-methyl d-aspartate epsilon [*Danaus plexippus*]	0	94	N
IR1	196	[c]	ADR64688 putative chemosensory ionotropic receptor 1 [*Spodoptera littoralis*]	7e-92	70	N
IR1.2[a]		[c]	Not identified			N
IR2[b]	251	KF768721	No putative ionotropic receptor 60a.2 [*Danaus plexippus*]	2.41-7	30	N
IR7d.1[b]	258	KF768722	No putative ionotropic receptor 7d.1 [*Bombyx mori*]	1.59e-77	42	N
IR7d.2[b]	312	KF768723	No putative ionotropic receptor 7d.2 [*Bombyx mori*]	1.32e-64	38	N
IR7d.3[b]	177	KF768724	No putative ionotropic receptor 7d.3 [*Bombyx mori*]	4.19e-42	36	N
IR8a	895	[c]	AFC91764 putative ionotropic receptor 8a, partial [*Cydia pomonella*]	0	81	Y
IR21a	857	[c]	ADR64678 putative chemosensory ionotropic receptor 21a [*Spodoptera littoralis*]	0	82	Y
IR25a	918	[c]	AFC91757 putative ionotropic receptor 25a [*Cydia pomonella*]	0	88	Y
IR41a	168	[c]	ADR64681 putative chemosensory ionotropic receptor 41a [*Spodoptera littoralis*]	6e-98	86	N
IR60a	382	KF768725	NP_611901 ionotropic receptor 60a [*Drosophila melanogaster*]	4e-38	28	N
IR64a	237	KF768726	NP_647962 ionotropic receptor 64a [*Drosophila melanogaster*]	3e-34	31	N
IR68a	273	KF768727	ADR64682 putative chemosensory ionotropic receptor 68a [*Spodoptera littoralis*]	2e-162	83	N
IR75d	222	[c]	ADR64683 putative chemosensory ionotropic receptor 75d [*Spodoptera littoralis*]	3e-97	88	N
IR75p	567	[c]	ADR64684 putative chemosensory ionotropic receptor 75p [*Spodoptera littoralis*]	0	89	N
IR75p.1	351	KF768728	AFC91755 putative ionotropic receptor 75p, partial [*Cydia pomonella*]	0	81	N
IR75p.2	131	[c]	ADR64684 putative chemosensory ionotropic receptor 75p [*Spodoptera littoralis*]	3e-12	39	N
IR75q.2	554	[c]	ADR64685 putative chemosensory ionotropic receptor 75q.2 [*Spodoptera littoralis*]	0	89	N

IR76b	544	[c]	ADR64687 putative chemosensory ionotropic receptor 76b [*Spodoptera littoralis*]	0	84	Y
IR87a	642	[c]	ADR64689 putative chemosensory ionotropic receptor 87a [*Spodoptera littoralis*]	0	91	Y
IR93a	586	KF768729	AFC91753 putative ionotropic receptor 93a, partial [*Cydia pomonella*]	3e-85	68	N

[a] These genes were not identified from our transcriptomes.

[b] The information of these divergent HarmIR genes was obtained from Local Blastx results due to low identity and unavailable data in NCBI.

[c] [17].

HarmGR10 was previously identified HarmGR1.
