# Supplementary material for: Identification and characterization of three chemosensory receptor families in the cotton bollworm Helicoverpa armigera
Source: BMC Genomics. 2014 Jul 15;15(1):597. doi: 10.1186/1471-2164-15-597 (PMC4112213; doi:10.1186/1471-2164-15-597)
Supplement: Supplementary file 3 — Additional file 3: Table S3: Primers for RT-PCR experiments of H. armigera OR, GR and IR genes and calcium imaging analysis of H. armigera OR genes. (PDF 8 KB) [file 12864_2013_6286_MOESM3_ESM.pdf]

Table S3

purpose	Gene	Forward primer (5' to 3')	Reverse primer (5' to 3')
**RT-PCR**	OR1	TCCATCATCATCTGCTTCTGTGGC	TCACTTTTTATAAATATTCAGTAGC
	OR49	GGACGAGCGTCAGACCTCCG	CCAGGGTGTACACCGCGAAGACG
	OR50	GAGGCTGGTGATGTACAGCCAGGC	GTCTCCGAGTGGAGTTTAATCCC
	OR51	GTCTTTTCCCGATTAGCTACTC	GAAGTAGAATTTTGAAGTGTCCCC
	OR52	ATGCGGACGTTACGTGAAATTGG	GAAGGCAGTCACGCAAGCAGGGAC
	OR53	GCAACGCCATCGCTTTACATAC	CGGGTATGTTACCACAAATTCACC
	OR54	CCTGGCGAAACTTATGGATTGAG	GTGGCATGATTCCTGACATCCC
	OR55	CTGGCTATGTCGGAGGTATTATCG	GCCAACAGTACAAAAACAGCTGCG
	OR56	CAGTCGTTTATATTGTCATCAC	CGAAAGAAGTATACATCAACCCAGC
	OR57	GCCAGTCTAAATAACATCATCATGC	CATGAGGACAGGGCCCAAG
	OR58	CCGTAACGTGCAGAAGTCTATGAG	GAATAAGACAAAGTGATGACTC
	OR59	CCCATTCCGCTACCCCTTAGCCTAC	CTATTCGTGTCGAAATTGAAGC
	OR60	ATGGGTGTACTAGTTCGTAACGC	TTACTCTTCAATAACTTGCTGTAAC
	GR1	ATGGAGTCTCAGTTTTGCCGTGATC	AGCTTATGGTTTCAAGGG
	GR2	ATGATATTGCGATCCATTCGACG	TTATTTATATTGGTCGGTGCTTTAC
	GR3	GTCGTAAACTGAATAGATACGC	GATAATATCATTAGAAATGTGCG
	GR4	GAGCATGATATCTGCTACTGGCG	CGACGCCATACTTGAATTTGTAGG
	GR5	CCTGATAGGTCAGTTTATTATGGC	CAAAGGAGTGTCAGGGTCACAGTC
	GR6	TCATTGGCCAGATGTTTATGGCAG	CAAAAGCGATAGTATGTGTTCAC
	GR7	ATGTATTTCTTCATTGGATTTATG	TATTTTCGCCAAGCACTGGTTTAGTC
	GR8	GATGTTGCTCTCGGCGGTATTATC	CCCGCTGAGGGCGATGTAATCTTTG
	GR9	CTCAGCTGTCAACGATGCTCTCGCG	CACCACTAGCCGAGGCTCCCCGC
	IR1	GGACACCACTAGTGCTGCTCG	CCAAAATTGTACACGTCGGTCA
	IR1.2	TACAAATATTATCGACGGTGGGA	ATCGGTAATCGTGGAAACATATCT
	IR2	AATGCTGTGATGACGACAATGA	TGTATAAAATGGCGCCAAATG
	IR7d.1	CAAGAATGCGACGAACTGAAAA	CTCCGGATACAGGTTCTTGAAAC
	IR7d.2	CAAGTCTCAAGATGATGACGCC	CCACACCACTTTGGAGGTAGAA
	IR7d.3	GAAGCAGTAACGAAAGTAGACGGT	TCTTCAAAATCTGAACATAGGGCT
	IR8a	CAGGGGATTGTCTGATGCTTC	GTCCACGGTGGGTTATGAAAA
	IR21a	CTGGGTAAGCAGCCAAAGAAC	TACAACTGGTTGACTAGCACCCA
	IR25a	CAAATCACTACTGGACTCTGGCA	TAAATTCCATATAGCTCCTGCCG
	IR41a	AAGCATGGCATACAGTATTGAAAA	GCAGTAGACCGGATTGATGGA
	IR60a	AATGGAACGGAGGTTTGAAGAA	CAGAATACCAAACCAAGCTCTTGA
	IR64a	TTTGGATAGCGACTTGAAGGC	AACGCAACACTTGTGTCGATG
	IR68a	CCAGCCCCAAGAGTTACCATT	GTTATCGTAGTAAGCGTACACCCCT
	IR75d	AGAACTCCGGCTACCGGGAG	CCAGCAGGGTCAGGGAGATG
	IR75p	TACCACGAGAGAGCATCTGCCT	TTAGAATACAGATCAGCGATCATGC
	IR75p.1	TGCTGGGATGCTCTATATTTCTGTA	AAGCCGCGTAGAGAATCGTTAG
	IR75p.2	CGTGGTATCAGACATAGAGCGTTT	TCTTTAGCTTCCCATTTCGCAG
	IR75q.2	CGATTTTTCGAACCAACCTCTT	CCCAAGTGTTGGCAAAAATGTA
	IR76b	TTATGCCGACATTGATAGCGTA	CCCTCGTGAAGAATGTTATGCA
	IR87a	GCTGTCCTTTGGTGGCTTATG	CCCCAATTTTCCAAAATATCTGA
	IR93a	AATGACACCCTTGCTGACACA	AATGGCAGCAGGAACAACAGT
**Calcium imaging**			
	OR13	GGGGTACCATGAAAATTCTATCAGACGGTTC	TCCCCGCGGTCACTGTTCTTCTTCTGCAACT
	OR51	CCCAAGCTTATGGCAGGGTTGCTTGATT	GCTCTAGATTACATACTGCGTAAGAAGGTGAA

Note: Restriction sites are underlined.
